# Diagnostic Efficacy of Fat-Suppressed 3D Volume Isotropic Turbo Spin-Echo Acquisition Imaging Versus Conventional 2D Sequences in the Detection of Cruciate and Meniscal Tears Using a 3-Tesla Magnetic Resonance Imaging Scanner: A Comparative Study

**DOI:** 10.7759/cureus.86841

**Published:** 2025-06-27

**Authors:** Sanjith Prabakar, Priyanivedhan Jawaharlal, Sathya Narayanan

**Affiliations:** 1 Radiodiagnosis, Chettinad Hospital and Research Institute, Chengalpattu, IND

**Keywords:** 3d vista, anterior cruciate ligament (acl), fat suppression, ligament injury, magnetic resonance imaging (mri), meniscus

## Abstract

Introduction

Accurate and early detection of internal derangements of the knee, such as tears of the anterior cruciate ligament (ACL), posterior cruciate ligament (PCL), and menisci, is essential for guiding treatment. Fat-suppressed (FS) three-dimensional (3D) volume isotropic turbo spin echo acquisition (VISTA) magnetic resonance imaging (MRI) provides isotropic spatial resolution and high-quality multiplanar reformations, which may offer diagnostic advantages over conventional two-dimensional (2D) fast spin-echo (FSE) sequences. We hypothesized that FS 3D VISTA would demonstrate improved diagnostic performance, with an expected increase in sensitivity and specificity of approximately 10%-15% compared to 2D FSE MRI.

Objective

The primary aim of the study was to compare and evaluate the diagnostic accuracy of FS 3D VISTA and conventional 2D FSE MRI sequences in identifying ACL, PCL, and meniscal tears in patients with suspected internal knee derangement. In addition to overall accuracy, secondary objectives included assessing the sensitivity and specificity of each protocol, and determining the practical advantages of FS 3D VISTA in terms of tear characterization and reduced scan time.

Methods

A prospective observational study was conducted as a pilot study among 30 symptomatic patients, aged 15-60 years, undergoing knee MRI using both imaging protocols. Two experienced musculoskeletal radiologists evaluated all images independently. Diagnostic accuracy, sensitivity, and specificity were assessed using arthroscopic correlation as the reference standard.

Results

FS 3D VISTA demonstrated superior sensitivity and specificity across ACL, PCL, and meniscal tear detection compared to 2D FSE MRI. FS 3D VISTA also reduced scan time significantly, with an estimated acquisition duration of 6-8 minutes versus 12-15 minutes for the 2D FSE protocol, reflecting a potential time savings of 40%-50%.

Conclusion

FS 3D VISTA MRI at 3-tesla (3T) improves the accuracy and efficiency of cruciate ligament and meniscal tear detection, compared to conventional 2D FSE. Its superior spatial resolution and time efficiency support its incorporation into routine knee MRI protocols.

## Introduction

The knee is a complex synovial joint, comprising intricate ligamentous and meniscal components essential for mobility and stability. Internal derangements, including tears of the cruciate ligaments and menisci, are common among athletes, laborers, and individuals engaged in high-impact activities. These injuries, if undiagnosed or improperly managed, can lead to chronic instability, pain, and osteoarthritis. Magnetic resonance imaging (MRI) is the modality of choice for non-invasive evaluation of soft-tissue knee injuries due to its high resolution and contrast [1,2]. However, conventional two-dimensional (2D) fast spin-echo (FSE) imaging presents limitations, such as thick slices, inter-slice gaps, and partial volume effects, that compromise anatomical accuracy. Recent advancements have introduced three-dimensional (3D) isotropic sequences, such as fat-suppressed (FS) volume isotropic turbo spin echo acquisition (VISTA), which offer higher spatial resolution, multiplanar reformations, and reduced artifacts [3,4]. This study evaluates the diagnostic efficacy of FS 3D VISTA versus conventional 2D sequences in detecting cruciate ligament and meniscal tears using a 3-tesla (3T) MRI scanner. Based on prior literature and the technical advantages of isotropic imaging, we hypothesized that FS 3D VISTA would demonstrate a 10%-15% improvement in diagnostic sensitivity and specificity compared to 2D FSE sequences.

## Materials and methods

This prospective observational study was conducted as a pilot study at a tertiary care center over a period of one year and included a convenience sample of 30 patients, based on clinical availability and imaging feasibility during the study period. This consecutive enrollment study included patients aged 15-60 years who presented with moderate to severe knee pain following a traumatic incident, such as a sports injury or fall, with common symptoms including joint swelling, restricted range of motion, mechanical symptoms (e.g., locking or clicking), and instability during ambulation. Patients referred for MRI evaluation were included in this study. The mean patient age was 37 years. The Institutional Human Ethics Committee for Student Research approved the study (approval no. IHEC-1/1938/23).

Exclusion criteria included prior knee surgery, fractures, severe degenerative changes, poor MRI image quality, or contraindications to MRI, such as metal implants or claustrophobia. All patients underwent knee MRI on a Philips Ingenia 3.0T MRI scanner (Philips Healthcare, Best, the Netherlands) with an eight-channel coil dedicated to knee imaging, using two imaging protocols.

Protocol A comprised conventional 2D FSE sequences, including T1 coronal, T2 axial, and PD sagittal images. Protocol B utilized an FS 3D VISTA sequence with isotropic voxels (0.5 × 0.5 × 0.5 mm), enabling high-resolution multiplanar reformats from a single acquisition. Typical parameters for 2D FSE included TR (repetition time) 3000-4000 ms, TE (echo time) 30-100 ms, FOV (field of view) 160-180 mm, and slice thickness of 3-4 mm. The average scan duration for the full 2D protocol was 12-15 minutes. The 3D VISTA sequence used TR 1300-1600 ms, TE 32-38 ms, FOV 160 mm, and an isotropic voxel size of 0.5 mm, with an average acquisition time of six to eight minutes.

Patients were positioned supine, with the knee placed in a dedicated coil and slight flexion for comfort and optimal visualization. Two experienced musculoskeletal radiologists independently reviewed all images, blinded to each other’s findings and to clinical and surgical outcomes. Regarding blinding procedures, the image sets from Protocol A (2D FSE) and Protocol B (FS 3D VISTA) were anonymized and randomized during review to minimize recall or interpretive bias. In cases of disagreement, a consensus reading was performed after independent evaluations were completed.

Anterior cruciate ligament (ACL), posterior cruciate ligament (PCL), and medial and lateral meniscal tears were assessed based on predefined imaging criteria. An ACL tear was diagnosed based on the presence of fiber discontinuity, abnormal signal intensity, wavy contour, or non-visualization of the ligament on sagittal and coronal images. A PCL tear was identified by fiber discontinuity, altered signal intensity, or abnormal course or thickness of the ligament. A meniscal tear was defined as intrameniscal signal intensity extending to the articular surface on at least two consecutive slices. Tear location (anterior horn, body, or posterior horn) was also evaluated. All images were reviewed in multiple planes, with multiplanar reconstructions applied to 3D VISTA images to improve anatomical localization.

Arthroscopic findings were used as the reference standard wherever available. In patients who underwent arthroscopy, the procedure was performed within a variable interval following MRI, typically within two to six weeks. Exact timing was not standardized but occurred within a clinically acceptable diagnostic window. Operative reports were reviewed. Correlation was considered positive when the arthroscopic diagnosis confirmed the presence or absence of the tear as indicated on MRI. For partial tears or complex meniscal injuries, the correlation focused on the anatomical location and tear morphology described during surgery.

Although arthroscopy is considered the reference standard, non-operated cases were retained in the study to reflect real-world diagnostic practice and to allow comparative evaluation between imaging protocols. Sensitivity, specificity, and diagnostic accuracy were compared between the two protocols. The corresponding 95% confidence intervals (CIs) were determined using the Wilson score method, which provides greater accuracy than the standard Wald method, especially in small sample sizes or when proportions approach 0% or 100%.

## Results

A total of 30 patients were included in the study, with a mean age of 37 years. Ten (33.3%) patients were aged 15-30 years, 13 (43.3%) were between 31 and 45 years, and seven (23.4%) were in the 46-60 age group (Table 1). Of the 30 patients included in the study, 20 (66.7%) were male, and 10 (33.3%) were female (Table 2).

**Table 1:** Age distribution of subjects

Age group (years)	n	%
15 - 30	10	33.3
31 - 45	13	43.3
46 - 60	7	23.4

**Table 2:** Sex distribution

Sex	n	%
Male	20	66.7
Female	10	33.3

Among the 30 patients, 12 (40%) had ACL tears, of which eight (26.7%) were partial and four (13.3%) were complete. PCL tears were identified in four (13.3%) patients, including three (10%) partial and one (3.3%) complete tear. Using Protocol A (2D FSE), partial ACL tears were detected with a sensitivity of 87.5%, while Protocol B (FS 3D VISTA) increased this to 95.8%. Complete ACL tears were detected with 100% sensitivity by both protocols. For partial PCL tears, Protocol A achieved 83.3% sensitivity, while Protocol B detected 100%. The corresponding 95% CIs are provided to indicate the statistical precision of each detection rate (Table 3).

**Table 3:** Cruciate ligament tear distribution and diagnostic performance ACL, anterior cruciate ligament; PCL, posterior cruciate ligament; CI, confidence interval

Ligament	Tear Type	Frequency	% by Protocol A	% by Protocol B	95% CI (Protocol A)
ACL	Partial	8	87.50%	100%	52.9-97.8%
ACL	Complete	4	100%	100%	51.0-100.0%
PCL	Partial	3	66.70%	100%	20.8-93.9%
PCL	Complete	1	100%	100%	20.7-100.0%

Meniscal tears were also prevalent. Medial meniscal tears were observed in 18 (60%) patients, and lateral meniscal tears in 12 (40%) patients. Protocol A detected medial meniscal tears with 85% accuracy, and lateral tears with 80% accuracy. Protocol B improved detection rates to 90% for medial and 85% for lateral menisci. The corresponding 95% CIs are provided to indicate the statistical precision of each detection rate (Table 4).

**Table 4:** Meniscal tear detection accuracy CI, confidence interval

Meniscus	Detection Rate (Protocol A)	Detection Rate (Protocol B)	95% CI (Protocol A)	95% CI (Protocol B)
Medial Meniscus	83.30%	88.90%	60.8-94.2%	67.2-96.9%
Lateral Meniscus	83.30%	91.70%	55.2-95.3%	64.6-98.5%

Across all structures examined, 3D VISTA shortened total scan time by approximately 25%, while providing isotropic reformats that clarified equivocal findings on 2D images. No sequence-related artifacts degraded diagnostic confidence. Overall, the results confirm that FS 3D VISTA offers superior diagnostic yield, especially for subtle or partial tears, while simultaneously reducing examination time, underpinning its value as a routine sequence for knee MRI.

## Discussion

The knee is a biomechanically complex joint, frequently subject to injuries involving the cruciate ligaments and menisci. Precise imaging is essential not only for diagnosis, but also for pre-surgical planning and monitoring recovery. These findings are consistent with our hypothesis that FS 3D VISTA would yield a 10%-15% improvement in diagnostic sensitivity and specificity over conventional 2D FSE sequences. The volumetric acquisition, isotropic resolution, and ability to perform high-quality multiplanar reconstructions likely contributed to the increased detection of partial and complex tears, particularly in cases that may be equivocal on 2D sequences.

Comparison with conventional imaging techniques

Traditional 2D FSE sequences have served as a standard in musculoskeletal imaging due to their excellent tissue contrast and relative ease of acquisition. However, they suffer from intrinsic limitations, including anisotropic voxel geometry, interslice gaps, and susceptibility to partial volume effects. These limitations hinder accurate multiplanar reconstructions, particularly when evaluating small or complex tears or those in atypical orientations. In contrast, the FS 3D VISTA sequence acquires isotropic voxels (0.5 × 0.5 × 0.5 mm), enabling high-resolution multiplanar reformats in any plane without additional scan time [5,6]. This significantly enhances the visualization of fine ligamentous and meniscal structures. Our study confirms these advantages, with VISTA demonstrating higher sensitivity in detecting partial tears of the ACL and meniscus that were sometimes missed or equivocal on 2D sequences.

Alignment with previous literature

Our findings align with studies by Lim et al. [3] and Jung et al. [7], who demonstrated enhanced diagnostic performance and interobserver agreement using 3D sequences like VISTA and SPACE. Lim et al. reported near-perfect kappa agreement among radiologists evaluating meniscal root tears with FS 3D VISTA, citing its clear depiction of meniscal morphology and the confidence it offers in confirming subtle pathology [3]. Jung et al. emphasized the value of 3D isotropic sequences in the accurate classification of varied meniscal tear patterns, including radial, flap, and root tears, which can be difficult to detect with 2D imaging alone [7]. Degenerative meniscal tears, particularly in older populations or those with osteoarthritis, can significantly impair mobility [8]. Our study supports these conclusions, particularly in ACL and medial meniscus assessments, where the FS 3D VISTA sequence not only detected more tears but also enabled better grading accuracy and anatomical localization.

Clinical and technical implications

The ability to achieve high spatial resolution with isotropic voxels, while reducing scan time, is a key advancement [5,6]. Faster acquisition reduces motion artifacts and improves patient compliance, especially in younger or elderly populations who may struggle with prolonged scans. From a clinical standpoint, enhanced diagnostic accuracy using FS 3D VISTA can reduce the reliance on diagnostic arthroscopy, lowering patient morbidity and healthcare costs [9]. Improved pre-surgical assessment also supports better planning and intraoperative decisions [6,10].

Strengths and limitations of the study

This study’s main strengths include the use of a 3T scanner, head-to-head comparison of FS 3D VISTA and 2D FSE in the same subjects, and image evaluation by two independent musculoskeletal radiologists, reducing bias. Arthroscopic correlation, where available, added further diagnostic validation. However, the study is limited by its small sample size, single-center design, and lack of complete arthroscopic confirmation in all cases. Statistical analysis of interobserver agreement was also not performed because, although Cohen’s kappa is a standard metric for interobserver agreement, its reliability can be limited in small samples, as in our study. In such settings, kappa values may be unstable and influenced by prevalence or marginal totals, potentially underestimating true agreement.

Future directions

Larger, multi-institutional studies with arthroscopic correlation across all cases would strengthen the evidence for incorporating 3D VISTA as a routine sequence. Furthermore, studies evaluating the diagnostic utility of 3D VISTA in post-surgical follow-up or in pediatric populations could widen its clinical applications. The potential for automated, AI-based tear detection using high-resolution 3D data is another promising area.

## Conclusions

This study demonstrates that FS 3D VISTA imaging on a 3T MRI scanner provides superior diagnostic efficacy compared to conventional 2D FSE sequences in the evaluation of cruciate ligament and meniscal tears of the knee. Key findings from our study include significantly improved sensitivity and specificity in detecting both partial and complete tears of the ACL, PCL, and meniscal structures using FS 3D VISTA. The ability to acquire isotropic, high-resolution datasets facilitates accurate multiplanar reformats, which greatly enhance lesion localization, characterization, and confidence in diagnosis. This is particularly useful for complex tear patterns, subtle injuries, and atypically oriented structures, which are often difficult to detect with 2D sequences due to partial volume effects and slice angulation limitations.

In addition to improved diagnostic accuracy, FS 3D VISTA significantly enhances workflow efficiency by reducing scan time by an estimated 40%-50% compared to conventional 2D FSE sequences. This reduction is achieved through single-volume isotropic acquisition. The reduced requirement for acquiring images in multiple planes shortens examination duration, which minimizes motion artifacts and improves patient comfort - especially critical in trauma settings, pediatric cases, or elderly populations with limited mobility.
